# Immuno-impedimetric Biosensor for Onsite Monitoring of Ascospores and Forecasting of Sclerotinia Stem Rot of Canola

**DOI:** 10.1038/s41598-018-30167-5

**Published:** 2018-08-17

**Authors:** Lian C. T. Shoute, Afreen Anwar, Scott MacKay, Gaser N. Abdelrasoul, Donghai Lin, Zhimin Yan, Anh H. Nguyen, Mark T. McDermott, Manzoor A. Shah, Jian Yang, Jie Chen, Xiujie S. Li

**Affiliations:** 1 Department of Electrical and Computer Engineering, University of Alberta, Edmonton, AB T6G 2V4 Canada; 2 Department of Biomedical Engineering, University of Alberta, Edmonton, AB T6G 2V4 Canada; 3 National Institute for Nanotechnology, National Research Council, Edmonton, AB T6G 2M9 Canada; 4 Department of Chemistry, University of Alberta, Edmonton, AB T6G 2E9 Canada; 5 Department of Botany, University of Kashmir, Srinagar, 190006 J&K India; 6 InnoTech Alberta, Vegreville, AB T9C 1T4 Canada

## Abstract

Sclerotinia stem rot, caused by the fungal pathogen *Sclerotinia sclerotiorum*, is a destructive disease of canola and many other broadleaf crops. The primary inoculum responsible for initiating Sclerotinia epidemics is airborne ascospores released from the apothecia of sclerotia. Timely detection of the presence of airborne ascospores can serve as an early-warning system for forecasting and management of the disease. A major challenge is to develop a portable and automated device which can be deployed onsite to detect and quantify the presence of minute quantities of ascospores in the air and serves as a unit in a network of systems for forecasting of the epidemic. In this communication, we present the development of an impedimetric non-Faradaic biosensor based on anti-*S. sclerotiorum* polyclonal antibodies as probes to selectively capture the ascospores and sense their binding by an impedance based interdigitated electrode which was found to directly and unambiguously correlate the number of ascospores on sensor surface with the impedance response.

## Introduction

Sclerotinia stem rot is one of the most destructive diseases for canola^1,2^. Stem rot is caused by the fungal pathogen *Sclerotinia sclerotiorum*. The loss in the yield of canola seeds, when the conditions for the disease are favorable, can be higher than 50%^3^. Canola is an increasingly important crop in the prairie region of Canada. In Alberta, the production of canola seeds increased from about 3.6 million tons in 2009 to 5.3 million tons in 2011^4^.

*Sclerotinia sclerotiorum* is one of the most non-specific, omnivorous, and successful plant pathogens. Plants susceptible to this pathogen encompass 64 families, 225 genera, and 361 species of broadleaf plants that are cultivated throughout the world^1,2^. Infection of plants by the pathogen occurs mainly due to airborne ascospores. In canola, these airborne ascospores come into contact and adhere to the petals. The ascospore germinates when the infected petal falls into the crop canopy and lands on the leaves, stems, and branches^5^. It grows and then spreads to the leaf and stem tissues. The fungus grows in the stem to produce sclerotia which can then be dislodged during harvest and serves as the inoculum for the subsequent years.

Sclerotia are compact masses of hyphae with the ability to survive in the soil for more than five years^4–6^. Under moist conditions, a sclerotium germinates to produce either mycelium, which may infect plants, especially root tissues in direct contact with the sclerotium, or ascospore-producing apothecia. Each apothecium can produce and release more than 10 million ascospores into the air^6^, thereby contributing to the spread of the infection over a large area. Hence, the presence of apothecia in the canopy and airborne ascospores can serve as an early-warning system for the stem rot infection of canola.

The primary tool used to control Sclerotinia stem rot of canola is the application of fungicides^7^. In order to be effective, foliar fungicides need to be applied during the key stage of infection, that is, early flowering and before the appearance of symptoms in the crops. Systematic application of fungicides is unprofitable because the outbreak of sclerotinia incidence can vary greatly among fields and years.

Therefore, a number of forecasting systems have been developed to predict the risk of stem rot infection^8,9^. The methodology adopted for risk assessment includes recording the amount of continuous rainfall for a number of days, soil moisture and apothecium development, temperature, crop canopy development, crop rotation, and crop disease levels in the previous years. Additional tools such as testing petals for ascospores infection and weather-based forecasting maps have been adopted in Canada^10,11^. However, these approaches for risk assessments are time consuming, labor intensive as they require constant field testing, and yet may not predict the risk in a timely manner.

As airborne ascospores are the dominant source of the spread of stem rot infection in susceptible plants, methods that can directly detect airborne ascospores offer the best and most direct measure of the risk of crop infection. In the case of carrots^12^, where a detailed correlation between the presence of airborne ascospores and incidence of Sclerotinia rot carrot epidemic are available, the initial occurrence of the disease was observed 8 and 34 days following the detection of 9.5 and 2 ascospores per m^3^ of air, respectively. In recent years, quantitative real-time polymerase chain reaction (qPCR) has been developed as the method of choice for monitoring airborne ascospores by amplifying a selected segment of their DNA for detection and quantification^12–17^. Although qPCR has the sensitivity and selectivity to detect the presence of pathogens to a level as low as a single ascospore in the sample, it has a number of disadvantages in terms of cost and complexity of the method due to the simultaneous requirements of thermal cycling and fluorescence detection which renders the technique unsuitable for routine onsite field applications.

In this article, we report on the design and development of a biosensor based on anti-*S. sclerotiorum* antibodies as probes immobilized on interdigitated electrodes (IDEs) and sense the binding of the ascospores by label-free non-Faradaic impedimetric detection for sensitive and selective detection and quantification of ascospores. As devices based on label-free non-Faradaic impedance detection are amenable to microfabrication and miniaturization, our goal is to develop a low cost, miniaturized, and automated biosensor for onsite monitoring of the number of ascospores suspended in the air around the canola fields and serves as an early warning system for farmers to forecast and manage the outbreak of Sclerotinia stem rot epidemic of canola.

## Results and Discussion

### Antibody immobilization

**Figure 1:**
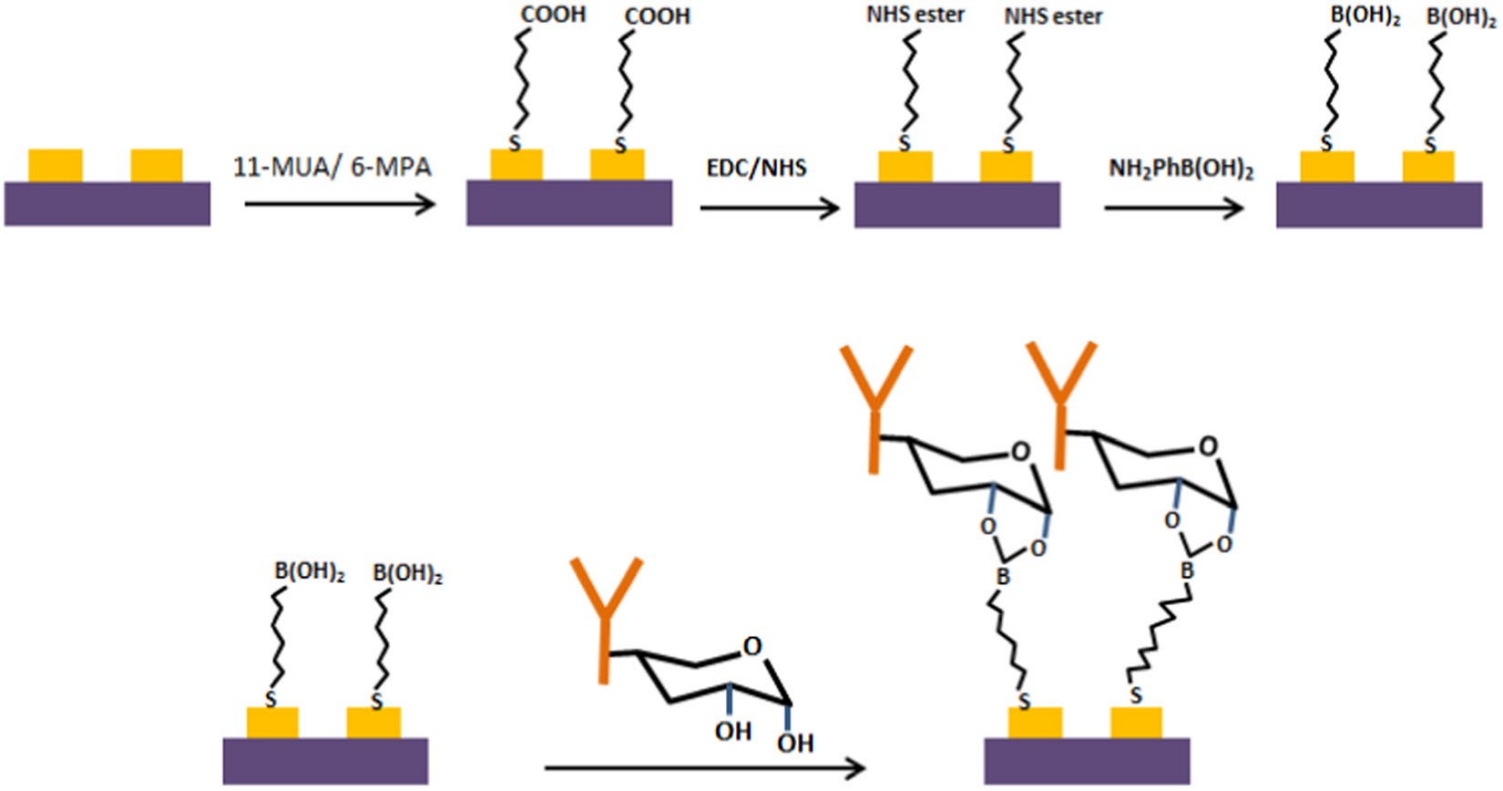
Schematic illustration of the functionalization of a gold IDE surface to covalently attach insulating self-assembled monolayer (SAM) and oriented immobilization of the anti-*S. sclerotiorum* antibodies by boronate ester conjugation.

One of the most important factors contributing to the development of a sensitive antibody-based biosensor is the ability to immobilize a high density of oriented antibodies covalently on the surface of the interdigitated electrodes (IDEs) so that the paratopes are free in the solution and available for efficient binding with the target antigens^18–20^. Sensitivity and stability, achieved in biosensors with well oriented covalently immobilized antibodies can be two orders of magnitude higher than biosensors with randomly oriented antibodies^21–23^. Figure 1 illustrates the surface functionalization steps and the process used to achieve oriented immobilization of anti-*S. sclerotiorum* antibodies on the surface of the IDEs. The first step in the functionalization of the gold surface of the IDE is the formation of a self-assembled monolayer (SAM) of alkanethiols with distal carboxylic acid end group. Alkanethiols with different chain lengths, viz 1-Mercapto-11-undecanoic acid (11-MUA) and 1-Mercapto-6-hexanoic acid (6-MHA) in aqueous ethanol solution with a molar ratio of 1:10 were used to form SAM to reduce steric crowding of the surface carboxylic acid end group. Treatment of the surface with EDC/NHS solution activates the carboxylic acid group by the formation of NHS ester which can efficiently react with the amino group of APBA to form an IDE with the gold electrode surface functionalized with boronic acid group.

Cyclic boronate esters are formed when boronic acid reacts with the 1,2- or 1,3-diol group of the carbohydrate moiety present in the fragment crystalizable region (Fc) of the antibody^18,21,24,25^. As the Fc region is located far away from the antibody binding sites and the boronate ester formation is specific to the carbohydrate moiety, the immobilization reaction afforded by boronic acid functionalized IDE provides well oriented antibodies with their paratopes facing the solution and readily available for efficient binding with the target antigens in the solution.

### Impedance spectra measurements and ascospores quantification

Electrochemical Impedance Spectroscopy is a powerful technique for investigating electrode/electrolyte interface as it provides rich information about the interface, interface structure, and interfacial electrode processes^26–34^. An impedance spectrum is obtained by applying a current or voltage excitation perturbation in an electrochemical cell and measuring the voltage or the current response as a function of the applied excitation frequency. Impedance is a measure of the opposition to the flow of current, arising from ion diffusion, electrode kinetics, redox reactions, and molecular interactions at the electrode surface, when an alternating excitation voltage is applied to the cell.

Depending on the presence or absence of redox active chemicals in the solution used for impedance measurement, a biosensor can be classified as either Faradaic or non-Faradaic^26–34^. In the Faradaic based biosensor, the factors contributing to the impedance are electrolyte resistance, double layer capacitance, interfacial electron transfer, and Warburg impedance. Whereas, in the non-Faradaic based biosensor such as the immuno-impedimetric biosensor described here, the contributions from the electrolyte resistance and the interfacial capacitances dominate the impedance of the system. In a non-Faradaic biosensor, the interfacial capacitances are sensitive to the probe-analyte binding occurring on the surface of the electrode and can be used for detection and quantification of target antigens. The total capacitance (C_tot_) of a sensor electrode can be considered as a combination of capacitances attributed to the SAM (C_SAM_), recognition layer (C_REC_), and double layer (C_DL_) connected in series^26–34^.

1 $$\frac{1}{{{\rm{C}}}_{{\rm{tot}}}}=\frac{1}{{{\rm{C}}}_{{\rm{SAM}}}}+\frac{1}{{{\rm{C}}}_{{\rm{REC}}}}+\frac{1}{{{\rm{C}}}_{{\rm{DL}}}}$$

and the impedance is related to capacitance as,

2 $${Z}_{C}=\frac{1}{j\omega C}$$

where, ω = 2π*f*, is the angular frequency and *f* is the applied frequency in hertz.

Due to the reciprocal nature of the relation, the total capacitance of the electrode/electrolyte interface is most sensitive to the changes in the magnitude of the smallest capacitance in the series. As C_DL_ in aqueous solution is normally very large value^35,36^, on the order of µF/cm^2^, a sensitive non-Faradaic impedance biosensor should be designed such that the C_SAM_ is as large as possible, and C_REC_ as small as possible^26–34^. Therefore, surface modification and immobilization of the antibodies play critical role in the development of a sensitive impedance based non-Faradaic biosensors.

**Figure 2:**
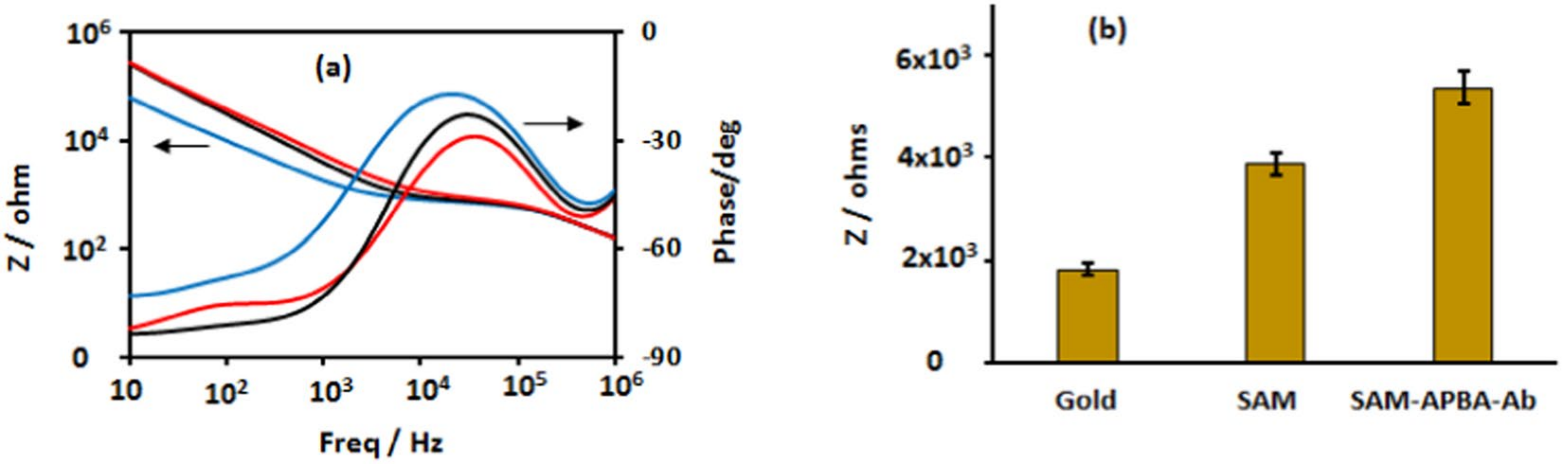
Impedance spectra, the plot of impedance magnitude and phase versus frequency for IDE gold electrodes **(a)**: (blue) bare gold electrode before modification, (black) after SAM modification, and (red) after anti-S. sclerotiorum antibody immobilization. Plot of impedance magnitude **(b)** determined at 1000 Hz for an IDE after different stages of surface modification.

Figure 2 shows the impedance spectra, the plots of the impedance magnitude and phase versus frequency, of the applied sinusoidal excitation potential (10 mV at 0 V DC) obtained for the IDE before modification (bare gold), after modification with SAM, and subsequently after immobilization of anti-*S. sclerotiorum* antibodies. It is known that the impedance magnitude of a non-faradaic impedance spectrum is dominated by interface capacitance, solvent resistance, and dielectric capacitance in the low, intermediate, and high frequency region of the spectrum, respectively^37,38^. As presented in Fig. 2, these regions of the spectrum are observed in the impedance spectra as expected. It is important to note that the capacitance of the system, as expected, is better represented by a constant phase element as indicated by the magnitude of the phase angle in the low frequency region. As shown in Fig. 2, modification of the bare gold IDE by SAM led to a huge increase in the impedance, as expected for capacitances connected in series with the double layer, because the SAM layer is composed of low dielectric alkane chains^39^. A further increase in impedance, albeit a smaller change was observed in the subsequent modification of the IDE surface with APBA and anti-S. sclerotiorum antibodies. To illustrate the observed impedance change following different stages of the IDE surface modification, the magnitudes of the impedance recorded at 1000 Hz (in Fig. 2a) are plotted in Fig. 2b.

The impedance change observed upon conjugation of APBA and anti-S. sclerotiorum antibody to the covalently attached SAM as displayed in Fig. 2 indicates that the fabricated sensor has the sensitivity to detect the binding of antigens to the surface immobilized antibodies. In the affinity based biosensors, a major source of interfering noise comes from nonspecific binding of biomolecules present in the solution. Although, the nonspecific binding on the sensor surface can be greatly reduced by the formation of SAM layer on the IDE^40^, further treatment of the surface with reagents such as a blocking agent is required to minimize their effects. In our biosensor, the IDE after surface modifications and anti-*S. sclerotiorum* antibody immobilization was incubated with 2% BSA solution to minimize nonspecific binding prior to treatment with the target ascospore solution.

Since the selectivity and specificity of an impedimetric biosensor depend solely on the affinity of the probe antibody to the target antigen, and as the transducer generates an output signal to any particles, pollens, fungi or bacteria that attached/binds to the antibody, the chances of false positive signal exist unless the antibody has low or no affinity these adventitious materials. The anti-*S. sclerotiorum* antibody used in this study was produced and purified by Cedarlane Labs (Burlington, ON, Canada, https://www.cedarlanelabs.com/). The specificity against *S. sclerotiorum* was confirmed by ELISA method. Specificity test perform in our lab showed that the polyclonal antibody has high affinity for the target antigen, *Sclerotinia sclerotiorum*, as expected and encouragingly displayed little or no affinity for the fungus, *Leptosphaeria maculans*, which is responsible for blackleg, another devastating fungal disease of canola. This antibody was intended to be used as a probe for determining the biosensor perimeters for Sclerotinia stem rot disease in laboratory study. The specificity and the possible cross reactivity of anti-*S. sclerotiorum* antibody with fungi commonly found in crop fields have been investigated by a number of groups^41–44^. Jamaux and Spire^41,42^ reported that anti-*S. sclerotiorum* polyclonal antibodies cross react with closely related species such as *S. trifoliorum* and *S. minor*, but do not cross-react with 30 other fungi which could possibly be found in different crop fields such as canola, wheat, sunflower, lettuce, soybean, etc. However, according to Bom and Boland^43^, the cross-reactivity among *Sclerotinia* spp. is not anticipated to be a significant source of error in the field because *S. homoeocarpa*, *S. trifoliorum and S. minor* are not typically found in the phyllosphere of canola due to their lack of spores, their host range, or both. The cross-reaction to *Botrytis cinerea*^41,42^ may cause problem in the polyclonal anti-*S. scleroriorum* antibody application. To overcome the cross-reactivity problem two approaches could be adopted to improve the antibody specificity for field application viz to perform absorption of *S. sclerotiorum* antigen with polyclonal anti-*B. cinerea* antibody as described by Jamaux and Spire^41^ or to develop monoclonal antibodies.

**Figure 3:**
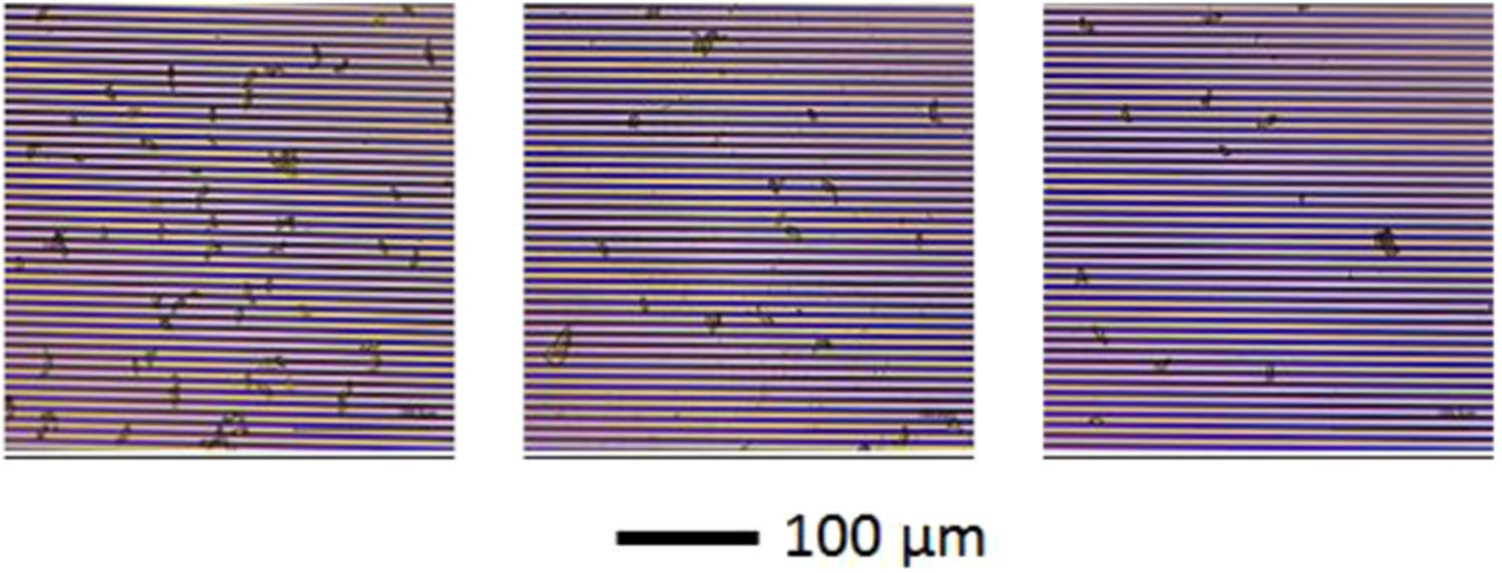
Optical images of ascospores selectively captured by immobilized anti-*S. sclerotiorum* antibodies on SAM modified IDE surface. Each 3 mm × 3 mm IDE was incubated, using PDMS mask with square wells, with a 50 µL solution containing a suspension of a desired concentration of ascospores in nanopure water. The number of ascospores on the surfaces i.e. ascospores/cm^2^ are **(a)** (1.1 ± 0.1) × 105, **(b)** (4.4 ± 0.4) × 104, and **(c)** (2.5 ± 0.3) × 104. Scale bar equals 100 µm.

Figure 3 shows the optical microscopy images of the ascospores selectively captured from ascospores in solution, via antigen-antibody affinity binding, by the immobilized antibodies on the surface of the IDE. It is important to note that the ascospores captured by the immobilized antibodies on the surface of the IDE are unaffected by repeated washing, indicating suitability of the boronic ester bond for antibody immobilization^18,21,24,25^. As the ellipsoidal shaped ascospore has size in the range, 4–6 µm × 9–14 µm^45^, a digital optical microscope was used in this work as a technique to verify the efficacy of the protocol used to capture ascospores by the immobilized antibodies.

The microscope images were also used for determining the number of ascospores captured on the IDE surface and to correlate their contributions to the impedance response. As shown in Fig. 3, the estimated number of ascospores in the representative images correspond to about 9700, 4000 and 2300 ascospores captured on the surfaces of 3 mm × 3 mm IDEs in the sensor chip. In this work, the numbers or concentrations of suspended ascospores in the solution were determined by using a hemocytometer. From the concentration of ascospores in the solution and the observed number ascospores captured on the surface of the IDE, we have determined that the antibody-target antigen binding proceeds with high efficiency and most of the ascospores present in the solution captured on the surface of the IDE.

**Figure 4:**
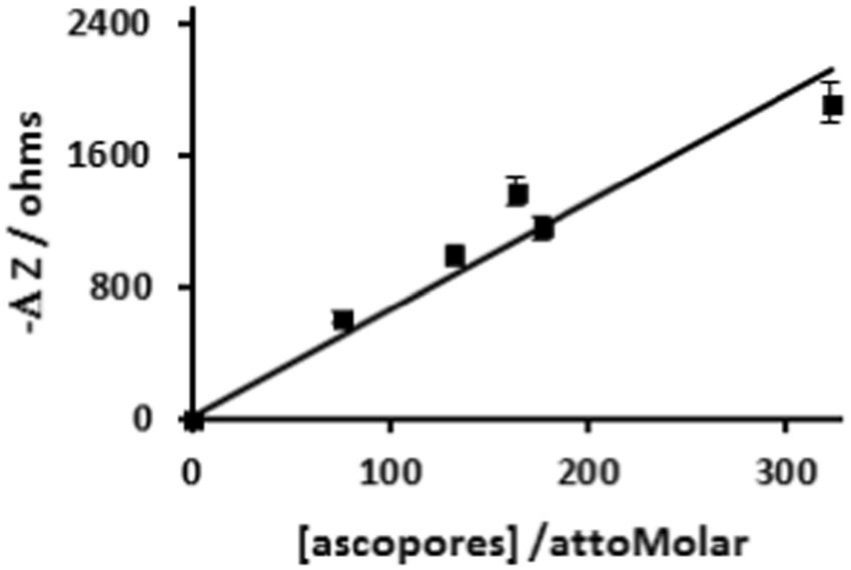
Plot of impedance change (ΔZ) versus ascospore concentration [ascospore] in the incubation solution. The black line is the linear fit to the experimental data point (filled squares) represent the means of four replicate experiments and error bars are RSD (n = 4). Where, ΔZ = Z_Ab-Sp_ − Z_Ab_, and Z_Ab_ and Z_Ab-Sp_ are the magnitudes of the impedance measured at 1000 Hz before and after the ascospores have been captured by the immobilized anti-*S. sclerotiorum* antibodies on the surface of modified IDE.

A primary goal of the present work is to establish whether an impedance based non-Faradaic biosensor is suitable for sensitive detection of ascospores in solution and eventually to develop a viable technology for field applications suitable for remote onsite sensing and forecasting of Sclerotinia stem rot epidemic of canola. Figure 4 shows the plot of impedance change (ΔZ) versus ascospores concentration [ascospore] in the incubation solution.

The impedance change (ΔZ) at any given ascospore concentration was calculated from the impedance spectra of an IDE recorded before (Z_Ab_) and after (Z_Ab-Sp_) the capture of the ascospores by the immobilized anti-*S. sclerotiorum* antibodies on the surface of the functionalized IDE. Binding of the ascospores on the IDE surface led to a decrease in impedance magnitude, thereby yielding a negative value for the impedance change, ΔZ = Z_Ab-Sp_ − Z_Ab_, calculated from the impedance magnitude at the applied sinusoidal AC frequency of 1000 Hz. As demonstrated by the experimental data plotted in Fig. 4, the impedance magnitude decreases with increase in the number of bound ascospores on the surface of the IDE.

The change in the magnitude of impedance in a capacitive based sensor due to affinity binding of target antigens to the surface immobilized antibodies can arise from the dielectric properties of the target antigens, the displacement of water molecules due the target binding, and the change in the thickness of the recognition layer^26–34^. These changes contribute to impedance change via the capacitance of the recognition layer (C_REC_) in the biosensor.

A unique advantage of working with ascospores is their size^41^ (4–6 µm × 9–14 µm) which allowed us to confirm the selective capture of ascospores on the sensor surface using optical microscope (Fig. 3). Further, the optical images were used to estimate the number ascospores captured on the IDE surface which in turn is related to the concentration of ascospores in the incubation solutions and these concentration values were plotted as shown in Fig. 4. Hence, these results allowed us to obtain a direct and unambiguous correlation between the number of selectively captured ascospores and their impedance response.

The experimental data in Fig. 4 can be fitted with a linear regression (R2 = 93%) to yield a slope of 6.5 ohms/aM ascospores in the solution. The limit of detection (LOD) evaluated from the signal-to-noise ratio determined from the standard deviations of the negative controls was about 130 aM.

In the traditional convention for the expression of LODs for biosensors, the non-Faradaic biosensor developed in this work has a LOD of 7.8 × 10^4^ ascospores/mL. The performance of the biosensor compares well with the LOD of 7.4 × 10^4^ CFU/mL reported by Varshney and Li^46^ in 2007 for the detection of *E. coli* O157:H7 by impedimetric biosensor using IDE as the transducer. However, in the past few years a significant progress has been achieved in lowering the LOD, and the state-of-the-art IDE impedimetric biosensor reported by the same group in 2015 has LOD of 100 CFU/mL^47^ for the detection of *E. coli* with assay time as short as 1 hour. In spite of the general conception that IDE offers a better signal-to-noise ratio and sensitivity compared to the biosensors with traditional macro-electrode, it is interesting to note that LOD as low as 3 CFU/mL^48^ for the detection of *Salmonella Typhimurium* has been reported for impedimetric biosensors using macro-electrodes as the transducers with assay time as short as 45 minutes. The impressive LODs achieved by these state-of-the-art IDE impedimetric biosensors are comparable to that of the LODs attained by advanced PCR methods for *E. coli* detection^49^. It is however important to point out that caution should be exercised when comparing LODs reported for *E. coli* detection because of the bacterium propensity to exponentially multiply to form a colony of huge number bacteria in the nutrient rich medium. In fact, using nutrient rich media, Settu *et al*.^50^ has reported LOD of 7 cells/mL for the detection of *E. coli*. In this respect it will be useful to note that the ascospores, the pathogen of canola, can germinate and grow to produce mycelium enabling them to infect canola plants but cannot multiply like *E. coli* to form colonies of ascospores. These differences can have significant impact on the biosensor sensitivity if a monoclonal antibody with epitope specific to the ascospores is used in the detection of *Sclerotinia sclerotiorum*.

Although the LOD achieved in this work is significantly lower compared to the current methods such as qPCR used for monitoring airborne ascospores^12–17^, impedimetric biosensors have many advantages in terms of cost, miniaturization, automation, and onsite remote sensing capability. In addition, to the best of our knowledge this is the first report on an impedimetric biosensor for ascospores detection and thereby has huge potential for further improvements in sensitivity and LOD.

Even with the present level of performance, the impedimetric biosensor presented in this work could be used, and is being tested, for the forecasting of the outbreak of Sclerotinia stem rot, because airborne ascospores can be readily captured by high throughput ascospore traps. The threshold number of ascospore in the air which allows an 8-day advanced forecasting of the outbreak of Sclerotinia stem rot^12^ is about 9 ascospores/m^3^. By pre-concentrating the ascospores with a spore trap, the impedimetric biosensor describe in this work can be used for detection and forecasting of the Sclerotinia stem rot outbreak and serves as an early warning system for the management and control Sclerotinia stem rot of canola.

## Conclusions

The paper presents the development of a novel label-free capacitive biosensor for the detection and quantification of ascospores, a pathogen which causes canola stem rot, the most destructive disease for canola crop. The device was fabricated by a functionalization protocol designed to achieve oriented immobilization of the antibodies to attach covalently to the SAM modified surfaces of the gold electrodes of the IDE by using boronic acid chemistry. Oriented immobilization affords a high density of non-denatured probes available for binding with the target ascospores, and the high affinity of the antibodies for the ascospores provides the selectivity and specificity of the biosensor. The biosensor has been determined to have sensitivity and LOD for ascospores of 6.5 ohms/aM and 130 aM. The LOD of 130 aM or 7.8 × 10^4^ ascospores/mL obtained for the developed biosensor is much higher compared to the LOD of 3 CFU/mL reported for state-of-the-art biosensors for the detection of food pathogens. This indicates that the biosensor for ascospores detection has a huge potential for further improvement and also highlighted the need for the development of a monoclonal antibody with a high affinity for a specific epitope of the ascospores and possessing no cross reactivity with all other fungi or pollens present in the field where canola is grown. Based on the performance of the state-of-the-art biosensors, it is not unreasonable to expect the development of a biosensor optimized to a level with the capability to detect ascospores with LOD of 10 ascospores/mL and hence to provide a biosensor with the ability to forecast the outbreak of the Sclerotinia stem rot epidemic of canola.

## Materials and Methods

### Material and reagents

1-Mercapto-11-undecanoic acid 97% (11-MUA), 1-Mercapto-6-hexanoic acid 90% (6-MHA), N-(3-(dimethylamino)propyl)-N′-ethylcarbodiimide hydrochloride (EDC), N-hydroxysuccinimide 98% (NHS), 3-3-aminophenylboronic acid monohydrate 98% (APBA), Ethanol (100%), disodium hydrogen phosphate, monosodium hydrogen phosphate, and bovine serum albumin 98% (BSA) were purchased from Sigma-Aldrich Canada Co. (Oakville, Ontario) and were used without further purification. Ultrapure water (18.2 MΩ/cm) obtained from Millipore equipment (Mili-Q water) for sample preparation and washing.

Polyclonal anti-*S. sclerotiorum* antibody was produced by Cedarlane lab following a standard procedure from rabbits using *S. sclerotiorum* as the antigen. Ascospores of *S. sclerotiorum* were produced using a standard method (InnoTech Alberta accession #184) by planting sclerotia, generated from sliced carrot roots, into a wet sand and incubating at 10 °C until the sclerotia germinates to form apothecia. The ascospores released from the apothecia were harvested by trapping onto a filter paper disc by applying vacuum.

### Gold IDE sensor chip and Polydimethylsiloxane (PDMS) mask

**Figure 5:**
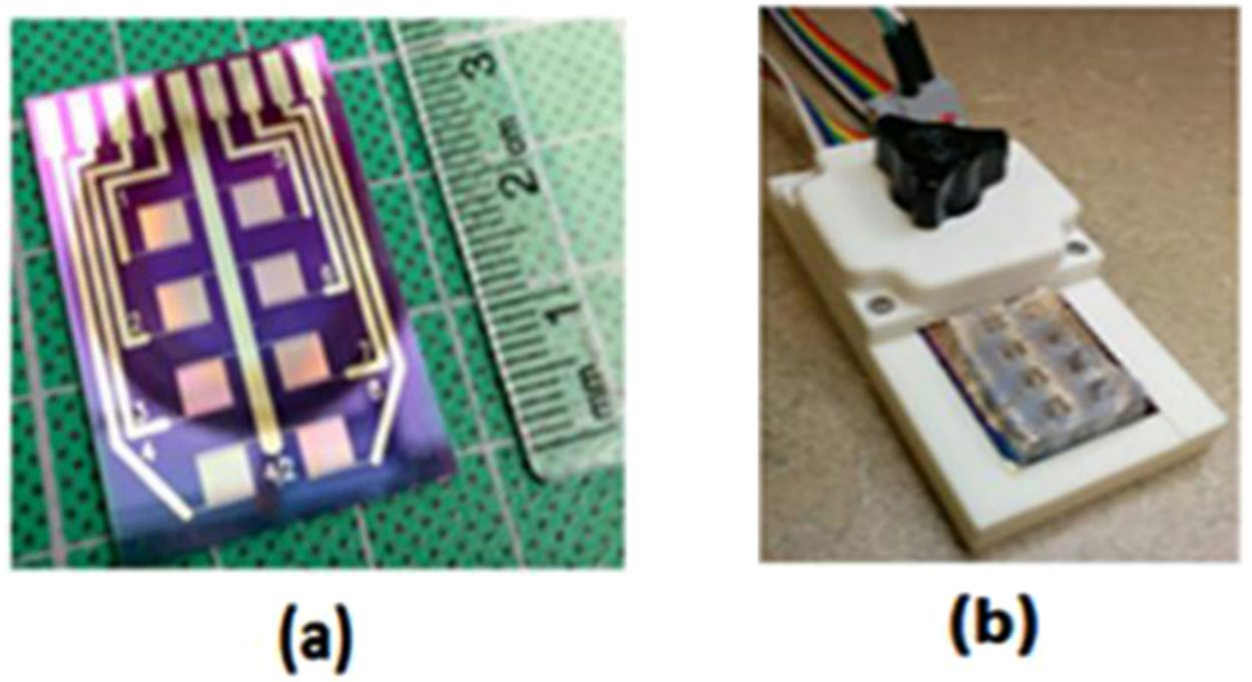
Sensor chip, PDMS mask, electrical contact pad, and electrical connector. The sensor chip has eight units of 3 mm × 3 mm square IDEs **(a)** and the matching PDMS mask has eight wells **(b)** to fit the IDEs and individually functionalize them. Each gold electrode finger or digit in the IDE has length, width, thickness, and gap of 3 mm, 3 µm, 110 nm, and 3 µm respectively.

A custom designed IDE sensor chip, with digit parameters optimized for nanoparticle-enhanced impedimetric sensors^51^, was fabricated on a silicon wafer with 500 nm thermal oxide following a standard photolithography process flow involving sputter deposition of chromium (10 nm) and gold (100 nm), photoresist and photomask pattern transfer followed by development, reactive ion etching and lift-off. Each of the sensor chips has eight 3 mm × 3 mm square IDEs with digit length, width, thickness, and gap of 3 mm, 3 µm, 110 nm, and 3 µm respectively. Images of a typical sensor chips is displayed in Fig. 5.

To facilitate functionalization of the IDE surface without affecting other areas of the chip, a PDMS mask, as shown in Fig. 5b, with eight units of 3 mm × 3 mm square wells, designed to fit the IDEs on the sensor chip was custom designed and fabricated in the lab. The PDMS mask allows independent and localized modification of each IDE on a sensor chip with any desired modifying solutions.

### Protocols for Surface functionalization

The chips used for surface functionalization were cleaned by sonicating for about 5 minutes each in acetone, isopropanol, Millipore water (MPW) and then drying with a stream of nitrogen. The chips were then exposed to Argon plasma (1 Torr Ar atmosphere, 18 W high RF) for 5 minutes to ablate any adsorbed organic materials on the surface. The freshly cleaned IDEs of the chip were functionalized in a sequence of successive reaction steps by submerging them in different solutions using the PDMS mask as presented in Fig. 5. After each functionalization reaction step, the IDEs were washed with the solvents used to prepare the solutions including ethanol, 10 mM PBS at pH 7.4 and/or MPW to remove any chemicals not covalently bound to the surface.

The affinity of thiol with gold was utilized to form an insulating self-assemble monolayer (SAM) with distal carboxylic acid group on the surface of the IDEs. The reaction was carried out by submerging the IDE overnight at 4 °C in a 50 µL of 10 mM 6-MHA and 1 mM 11-MUA in 95% aqueous ethanol solution. After washing thoroughly with ethanol and MPW, the SAM modified IDEs were submerged in a 50 µL of 0.1 M EDC and 0.1 M NHS aqueous solution for 20 mins. The IDEs were washed and submerged in a 50 µL of 52 mM APBA solution (10 mM PBS at pH 7.4) for 3 hrs. The boronic acid functionalized IDEs were submerged overnight in 50 µL of 5 µg/mL anti-*S. sclerotiorum* antibody buffered solutions (10 mM PBS at pH 7.4).

### Instruments

Electrochemical Impedance Spectroscopy (EIS) measurements were performed with a potentiostat/galvanostat SP-200 controlled by EC lab software from BioLogic Scence Instruments Inc (Knoxville, Tennessee). A custom built electrical contact pad and connector, as shown in Fig. 5b, was used to make electrical connections between SP-200 and the IDEs on the sensor chips. A PDMS mask with eight wells was used to submerse the IDEs of the sensor chip with 50 µL of 10 µM PBS at pH 7.4 for impedance spectra measurements. Impedance spectra were measured by applying 10 mV sinusoidal excitation perturbation at 0 V DC in the frequency range of 10 Hz to 1 MHz.

A digital optical microscope, VHX-700F from KEYENCE Canada Inc. (Mississauga, Ontario), was used for imaging and estimating the number of ascospores captured on the surface of the IDEs. A Hemocytometer and Motic AE 31 An inverted Biological Microscope (Carlsbad, California) was used to determine the number and concentration of ascospores in the re-suspended solutions.

### Data availability

The data files including raw data used to support the findings in this study can be obtained from the corresponding author upon reasonable request.
